# Great Wit and Madness

**Published:** 1888-07-07

**Authors:** 


### Great Wit and Madness.

Whether the old dictum about the alliance between great wit and madness, or Charles Lamb's neat phrase, "the sanity of true genius," is the nearer to the truth will always be a disputed point. Dr. Moreau, of Tours, wrote a book intended to prove that genius was a species of nervous disease; and though we do not hold with this very large statement, but, indeed, think that the highest genius can exist only when united to a perfect balance of the faculties, we must admit that some forms of genius, and these the most fascinating in their originality, invariably co-exist with a nervous system so sensitive that a very slight impetus from outward circumstance will mar its perfect organisation, and mark it, occasionally at least, with some of the characteristics of madness. Genius is insight, a vision of those meanings of natural facts and phenomena which the average comprehension misses. Genius, cultivated into art, may develop the tendency to seek for these super-sensual ideas to such a point that the true and perfect understanding of facts as they are, may be lost in exaggerated fantasies. Straining the spiritual sight occasionally produces moral myopia.

The poet Shelley is the most marked example we possess of this combined striving after the highest truths, and failure in the simplest duties of life, though Georges Sand and Alfred de Musset may claim a prominent place in the list. Shelley was filled with the most exquisite aspirations after the ideal life; he wanted everything—life, love, and liberty—in the subtlest essence, and the end of it all was that he completely lost his moral sense, and became as much the slave of passion as the grossest sensualist, with the additional misfortune of never for a moment doubting his perfect right to gratify his every caprice. Selfishness is bad at any time; but where selfishness decks itself in the iridescent robes of extreme sensitiveness it is exceptionally repulsive. Shelley's seeking after ideal emotions brought about his first wife's death, and enough has been recorded, both by himself in poetry and his friends in biography, to show that his second wife's life was far from being a happy one. She herself tried, after his death, to keep only the fairer side of his character before her own eyes and the world's, but one anecdote told of her betrays more than she would have liked.

Being advised to send her son to some school where he would be taught to "think for himself," she exclaimed impulsively, "No, not that; let him go where he will be taught to think like other people."

But there can be no doubt that the balance of Shelley's mind was far from perfect. His charities were as fantastic as his faults, and the very wisdom of many of his views on political and social questions makes all the more marked the lack of power to apply thought to action which showed itself in his life. He admits himself that he was the victim of a "nervous irritability, which, if not incessantly combated by myself and soothed by others, would leave me nothing but torment in life." His tendency to hallucination belongs also to disease. Though some critics set down his tales of visions, imaginary attacks by highwaymen, and the like, under the simple heading of "lies," the physical effect they had upon him proves that to him at least they were real. Yet in spite of his moral defects and intellectual follies Shelley's poetry is in a marked degree, if we except the crude boyish atheism of "Queen Mab"—noble in sentiment, more inspiring and ennobling to others than it proved to himself. How is this? Was the genius part of a nervous disease, or the disease an accident detrimental to the genius? It is difficult to say; and Shelley remains for all generations a bright example of the combination—fortuitous or inevitable, who shall decide!—of great wit and madness.

